# Esophageal cervical spondylosis complicated with cervical disc herniation: A rare case report

**DOI:** 10.1097/MD.0000000000030804

**Published:** 2022-09-30

**Authors:** Chaojun Zhu, Jianhong Tao, Songquan Mo

**Affiliations:** a Department of Orthopaedics, Santai County People’s Hospital, Mianyang, China.

**Keywords:** cervical intervertebral disc protrusion, dysphagia, esophago-cervical spondylosis, operation

## Abstract

Esophageal cervical spondylosis is rare in clinical practice, and the patients with cervical disc herniation are more rare.

**Patient concerns:**

A 56 year old male patient had dysphagia for 2 years, which was more obvious in the last month, and presented with pain and numbness in the right shoulder and upper arm.

**Diagnosis:**

The patient suffered from dysphagia. Gastroscope showed that the inner membrane of the esophagus was intact, chronic esophagitis, local smooth swelling, and no new organisms. DR shows a huge osteophyte in front of the cervical spine.

**Intervention:**

Anterior approach of cervical 4 and 5 anterior osteophyte resection, cervical 4/5 intervertebral disc resection, interbody fusion and internal fixation.

**Outcomes:**

Three days after operation, the dysphagia of the neck was significantly improved, and the numbness and pain of the right limb disappeared. The patient was very satisfied with the treatment.

**Conclusion:**

Anterior cervical anterior osteophyte resection, cervical disc resection, interbody fusion and internal fixation can effectively solve esophageal cervical spondylosis with cervical disc herniation.

**Lessons:**

Through the understanding of the disease, we can better understand the disease. It provides a treatment scheme for similar diseases.

## 1. Introduction

Esophago-cervical spondylosis is a disease caused by calcification and ossification of the anterior longitudinal ligament of the cervical spine, hyperosteogeny of the vertebral body and osteophyte formation, which oppresses the esophagus and then leads to poor swallowing.^[1,2]^ The causes of its formation include ankylosing spondylitis (AS), diffuse idiopathic skeletal hyperostosis (DISH).

## 2. Case report

**Figure 1.**
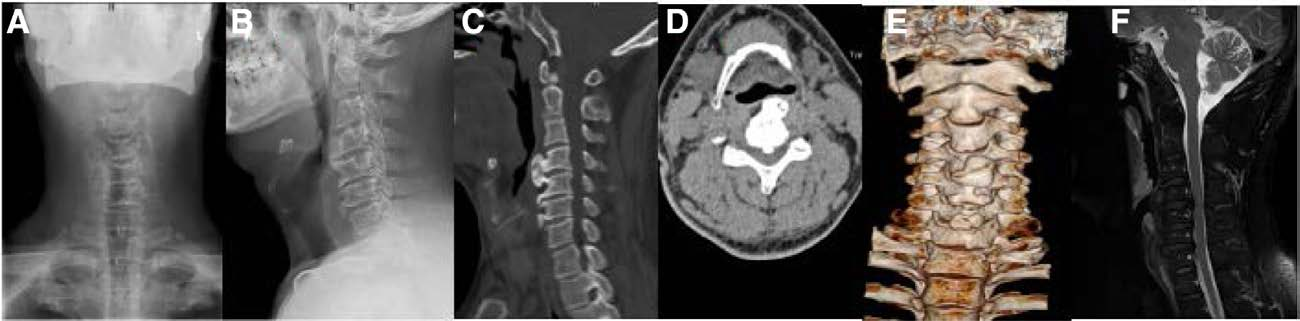
(AB) Anterior and lateral positions of cervical spine show huge osteophytes in front of C4 and 5 segments; (CD) CT of cervical spine showed anterior airway stenosis; (E) Three dimensional reconstruction of cervical spine; (F) C4/5 intervertebral disc protrusion can be seen on MRI of cervical spine.

The patient, male, 56 years old, was admitted with “dysphagia for 2 years, aggravated for 1 month, pain and numbness in the right shoulder and upper arm for 3 months”. Physical examination on admission: tenderness and percussion pain in the cervical 4/5 spinous process space, hypesthesia in the lateral arm of the right shoulder, and no significant hypesthesia in both lower limbs. Momentum diagnosis: neck flexion and extension are limited, right elbow flexion muscle strength is grade 4, left shoulder abduction, shoulder adduction, elbow flexion, elbow extension, wrist extension, wrist flexion muscle strength is grade 5. The right brachial plexus traction nerve test is positive. The straight leg raising test of both lower limbs was negative. Bilateral knee reflex and bilateral Achilles tendon reflex are normal, and bilateral Babinski sign (–). Dyspnea may occur when the neck is in an overextended position. X-ray of cervical vertebra shows that there are large osteophytes in front of cervical vertebrae 4 and 5 (Fig. 1A–B), and osteophytes in front of cervical vertebrae 5 and 6. MRI of cervical spine: the cervical 4/5 intervertebral disc protrudes to the right and compresses the cervical nerve root (Fig. 1E). CT scan of cervical spine showed that the osteophyte in front of cervical vertebra 4 pressed the esophagus and airway forward (Fig. 1C–E). The thickest part of the osteophyte is about 18 mm from the front edge of the vertebral body, and the airway is compressed and pushed forward. It can be inferred that the front esophagus is also compressed. In order to eliminate esophageal diseases, our department performed esophageal gastroscopy, which showed that the inner membrane of the esophagus was intact, chronic esophagitis, smooth swelling in some parts, and no new organisms were found.

Our department provides surgical treatment Scheme 1: anterior cervical 4 and 5 anterior osteophyte resection, cervical 4/5, 5/6, 6/7 intervertebral disc resection, interbody fusion and internal fixation. Advantages: solve the problem at one time and avoid possible secondary surgery. Disadvantages: the range of motion of cervical spine after operation decreases. Scheme 2: anterior approach of cervical 4 and 5 anterior osteophyte resection, cervical 4/5 intervertebral disc resection, interbody fusion and internal fixation. Advantages: solve the main symptoms of this time, and retain the mobility of cervical spine. Disadvantages: cervical surgery may be performed again. The patient’s cervical spine CT sagittal position showed that the anterior osteophytes of cervical segments 5, 6, 6, and 7 were not connected and bridged, and were not mature and stable, and the anterior osteophytes would continue to increase in the future. Therefore, it was suggested that the patient give priority to three segment anterior cervical discectomy and fusion (ACDF), and the patient approved and accepted the scheme.

**Figure 2.**
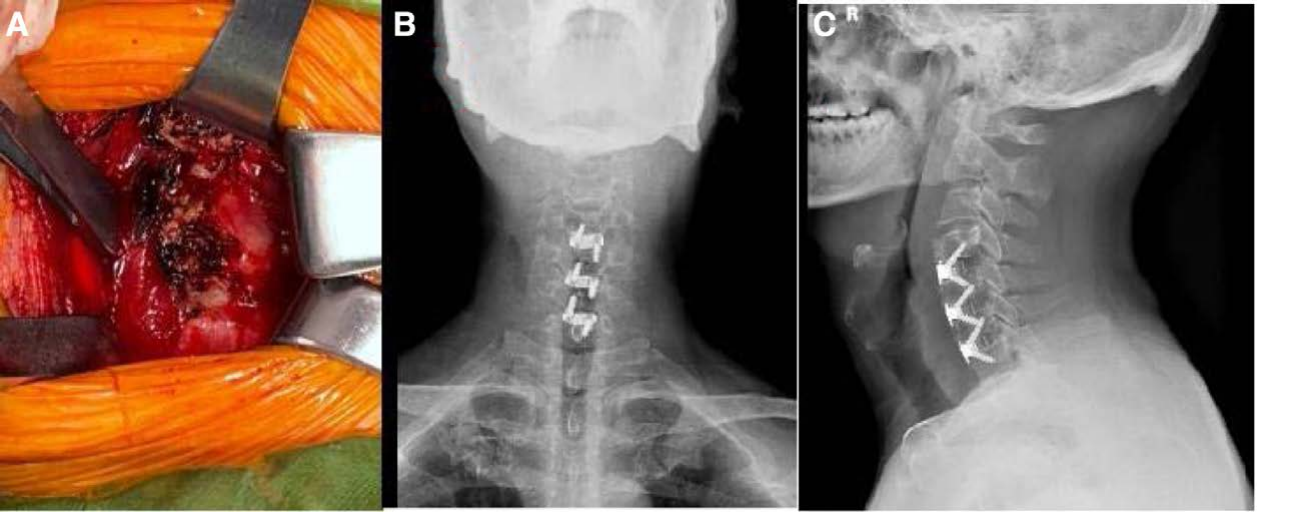
(A) shows the exposure of anterior osteophytes during operation; (BC) reexamination 2 days after cervical spine anterior and lateral position surgery 2 discussion.

**Figure 3.**
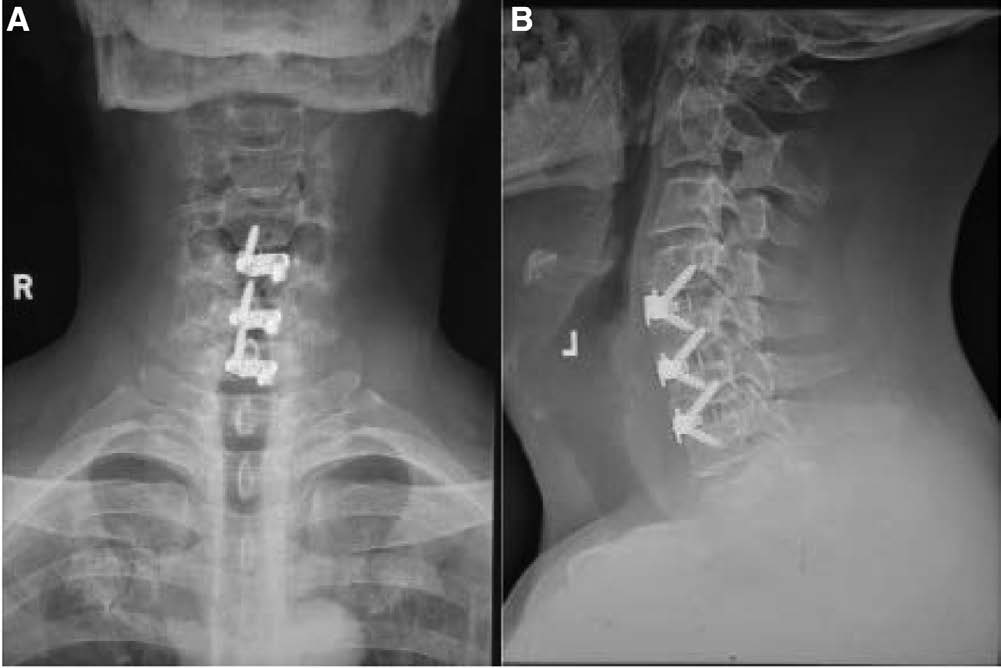
(AB) reexamination 2 months after anterior and lateral cervical spine surgery.

Operation process: general anesthesia after endotracheal intubation. The patient’s cervical spine should be properly extended in the supine position. Before operation, C-arm fluoroscopy was performed and the target segment was marked. A transverse incision of about 5 cm was made at the right dermatoglyphic area in front of the neck. The skin and subcutaneous skin were cut to the platysma and cut. After the platysma was secretly stripped to both sides of the head and tail, the medial edge of the sternocleidomastoid muscle was carefully identified, and the joint aponeurosis between the vascular sheath and the visceral sheath was cut, and the joint aponeurosis between the vascular sheath and the visceral sheath was entered from its gap. The sternohyoid muscle the thyrohyoid muscle is also pulled to the inside. When pulling it, pay attention not to damage the esophagus. After the exposure of the front of the vertebral body is completed (Fig. 2A), bipolar electrocoagulation is used to stop bleeding, and the wound is washed with physiological color. After exposing the osteophytes in front of the vertebral body, bite off the huge osteophytes in front with bone biting forceps, grind the drill to flatten the hyperplastic osteophytes in front, and then seal with bone wax to stop bleeding. The peanut gauze ball peeled off the anterior vertebral fascia, cut open the anterior longitudinal ligament in front of the vertebral body, use the Caspar spreader to open the exposed operation field, cut the anterior longitudinal ligament with a sharp knife oval, clean the intervertebral disc tissue with nucleus pulposus forceps, scrape the upper and lower cartilage endplates with a curette, remove the posterior longitudinal ligament at the level of c4/5 intervertebral space, and relieve the pressure in front of the dural sac and nerve root. The physiological lordosis of cervical spine and the height of intervertebral space were properly restored by using the spreader. After the size of the fusion cage is appropriate, the zero notch fusion cage is implanted into the intervertebral space one by one, the position is confirmed to be satisfactory under the perspective of the C-arm machine, and the screws are screwed into the upper and lower vertebral bodies of the fusion cage. The autologous bone fragments removed during decompression were filled next to the zero notch fusion cage. Fluoroscopy again confirmed that the zero notch fuser and screw length were satisfactory. After washing the wound and hemostasis, place a plasma drainage tube and sew it layer by layer. Three days after operation, the dysphagia of the neck was significantly improved, and the numbness and pain of the right limb disappeared. The patient was very satisfied with the treatment (Fig. 2B–C). Reexamination 2 months after anterior and lateral cervical spine surgery (Fig. 3(A–B)).

## 3. Discussion

### 3.1. Etiology of esophageal cervical spondylosis

Cervical spondylosis esophagus belongs to a rare type of cervical spondylosis. An epidemiological survey conducted by experts found that cervical spondylosis esophagus accounts for about 1.6% to 1.7% of cervical spondylosis. The main cause of this type of cervical spondylosis is the ossification of the anterior ligaments of the cervical spine and the bone compression formed by the hyperplasia of the vertebral body, resulting in esophageal compression and dysphagia.^[3,4]^ There are many reasons for ossification of the anterior longitudinal ligament and hyperosteogeny of the vertebral body, including diffuse idiopathic bone hypertrophy, ankylosing spondylitis, acromegaly, trauma, hypoparathyroidism, etc, of which the most common are diffuse idiopathic bone hypertrophy and ankylosing spondylitis.

### 3.2. Diagnosis of esophageal cervical spondylosis

Cervical spondylosis esophagus can be diagnosed after confirming the compression behind the esophagus on imaging and excluding other causes of dysphagia. Dr photographic examination or CT of cervical spine can clearly show the presence of huge osteophytes in front of cervical spine. Esophageal barium swallow test showed that When esophageal barium swallow test shows that the contrast agent passes through the osteophyte hyperplasia position, there were wavy indentations or smooth arcs on the posterior wall of the esophagus, while the mucosa of the esophageal wall was continuous without defects. Fiberoptic endoscopy can show that the posterior wall of the esophagus is swollen and the mucosa is normal. Therefore, the diagnosis of cervical spondylosis esophagus requires imaging data combined with clinical manifestations of patients, and it is necessary to exclude dysphagia caused by brain diseases and esophageal diseases, especially among the elderly. We encountered some cases of osteophyte hyperplasia in front of cervical vertebra in clinical work, but there was no case of dysphagia.

### 3.3. Differential diagnosis

DISH disease (diffuse idiopathic skeletal hyperostosis). Bazaz et al proposed the imaging diagnostic criteria of dish disease as follows: there are flowing water like ossification at the anterolateral edge of 4 or more consecutive vertebrae, and osteophyte formation at the junction of intervertebral disc; There was no obvious intervertebral disc degeneration, and the intervertebral space height was normal; There is no destruction, sclerosis or fusion of intervertebral joints and sacroiliac joint surfaces. The imaging data of this patient showed that there was no flow like ossification of more than 4 anterolateral vertebrae. AS tends to occur in young men. Ankylosing spondylitis is an immune disease. The sacroiliac joint space is blurred, destroyed and fused at the earliest time. When the lumbar vertebrae, thoracic vertebrae, and cervical vertebrae are dilated, the spine can form a typical “bamboo like” change, and ESR and C-reactive protein can increase during the active period. About 90% of patients are HLA-B27 positive. Differentiation of esophageal cancer. Barium meal examination of esophageal cancer showed mucosal interruption and destruction, filling defect, esophageal wall stiffness, etc. In addition, it also needs to be differentiated from pharyngeal diverticulum, laryngeal tumor, achalasia, and other diseases. Pharyngeal diverticulum may have the feeling of swallowing foreign bodies or obstruction, and produce the sound of angry water. With the gradual increase of diverticulum, dysphagia and food reflux may occur; Laryngeal tumors may have hoarseness; Achalasia may have paroxysmal dysphagia.

### 3.4. Mechanism of dysphagia caused by esophageal cervical spondylosis

Dysphagia is caused by the direct compression of esophagus by hyperplastic osteophyte at the anterior edge of cervical spine, the spasm and contraction of esophagus stimulated by pain, and the edema of tissue around esophageal wall stimulated by osteophyte. In addition, if the patient is in supine position, while the cervical osteophyte compresses the esophagus, it will also lead to tracheal stenosis and dyspnea. This patient has obvious airway stenosis (c4/5).

### 3.5. Treatment of esophageal cervical spondylosis

The early clinical symptoms are mild, which are mainly treated by liquid diet, oral nonsteroidal anti-inflammatory drugs, corticosteroids, and muscle relaxants. In the later stage, the clinical symptoms are serious.^[5]^ Through surgical treatment, the esophageal pressure can be directly removed and the mechanical compression can be directly relieved, but the specific surgical plan is not unified. Burkus^[6]^ believe that the osteophyte in front of the cervical spine is relatively stable, and only simple osteophyte resection is required, without internal fixation. Other scholars believe that after removing the proliferative osteophyte in front of the cervical spine, the intervertebral disc is unstable, and advocate bone grafting and fusion fixation after cervical disc resection.^[5,7]^ We believe that cervical hyperplastic osteophytes are mostly caused by degeneration and instability, resulting in hyperosteogeny and ligament ossification in order to rebuild stability. Removal of proliferative osteophytes will inevitably destroy the integrity of the anterior longitudinal ligament. Even if the cervical spine is relatively stable in a short time, cervical instability and spondylolisthesis will occur in the later stage. Therefore, bone grafting, fusion and internal fixation are still advocated.

## Author contributions

Chaojun Zhu produced the images used in the manuscript and wrote the manuscript. Jianhong Tao provided the patient data and performed the literature review. All authors have read and edited the manuscript and approved the version to be published. Conceptualization:Songquan Mo;Investigation:Jianhong Tao;Supervision:Songquan Mo;Writingoriginaldraft:Chaojun Zhu;Writing-review & editing: Songquan Mo.
